# Supplementary material for: Growth/Differentiation Factor 15 Promotes a Pro‐Regenerative Response in Chondrocytes Upon Cartilage Injury
Source: MedComm (2020). 2025 Nov 23;6(12):e70484. doi: 10.1002/mco2.70484 (PMC12640616; doi:10.1002/mco2.70484)
Supplement: Supplementary file 1 — Figure S1: Difference of syGDF15 concentrations in female and male patients. Figure S2: Results of the gene expression analysis of GDF‐15 and GFRAL after cartilage trauma. Figure S3: Heatmap presenting significantly upregulated (red) and downregulated (blue) genes after GDF‐15 stimulation as compared to untreated control hAC. “Ctrl” = unstimulated hAC; “Treat” = hAC stimulated with 100 ng/mL rhGDF‐15 for 48 h. Labeling at the right side describes gene names of proteins related to the glycerophospholipid metabolism. Figure S4: Additional findings of RNA‐seq analysis and confirmation of senescence‐associated genes by means of qRT‐PCR. Figure S5: Dose‐dependent effects of rhGDF‐15 on proliferation (alamarBlue) and migration (scratch assay) of hAC Figure S6: Influence of GDF‐15 on directed (Boyden chamber) and non‐directed (scratch assay) migration Figure S7: Anti‐inflammatory/immunomodulatory effects of rhGDF‐15. Table S8: List of primer and TaqMan gene expression assays used for qRT‐PCR. Figure S9: Influence of previous hyaluronidase digestion on concentrations of synovial GDF‐15. [file MCO2-6-e70484-s001.docx]

**Supplement**

**Figure S1: Difference of syGDF15 concentrations in female and male patients.**

Comparison of syGDF-15 concentrations in female and male OA patients (IOA and PTOA combined).

**Figure S2: Results of the gene expression analysis of GDF-15 and GFRAL after cartilage trauma.**

Gene expression analysis of (A) GDF15 and (B) GFRAL at 24 h, 7 d, and 14 d after *ex vivo* cartilage trauma.

**Figure S3: Heatmap presenting significantly upregulated (red) and downregulated (blue) genes after GDF-15 stimulation as compared to untreated control hAC.** “Ctrl” = unstimulated hAC; “Treat” = hAC stimulated with 100 ng/mL rhGDF-15 for 48h. Labelling at the right side describes gene names of proteins related to the glycerophospholipid metabolism.

**Figure S4: Additional findings of RNA-seq analysis and confirmation of senescence-associated genes by means of qRT-PCR.**

**
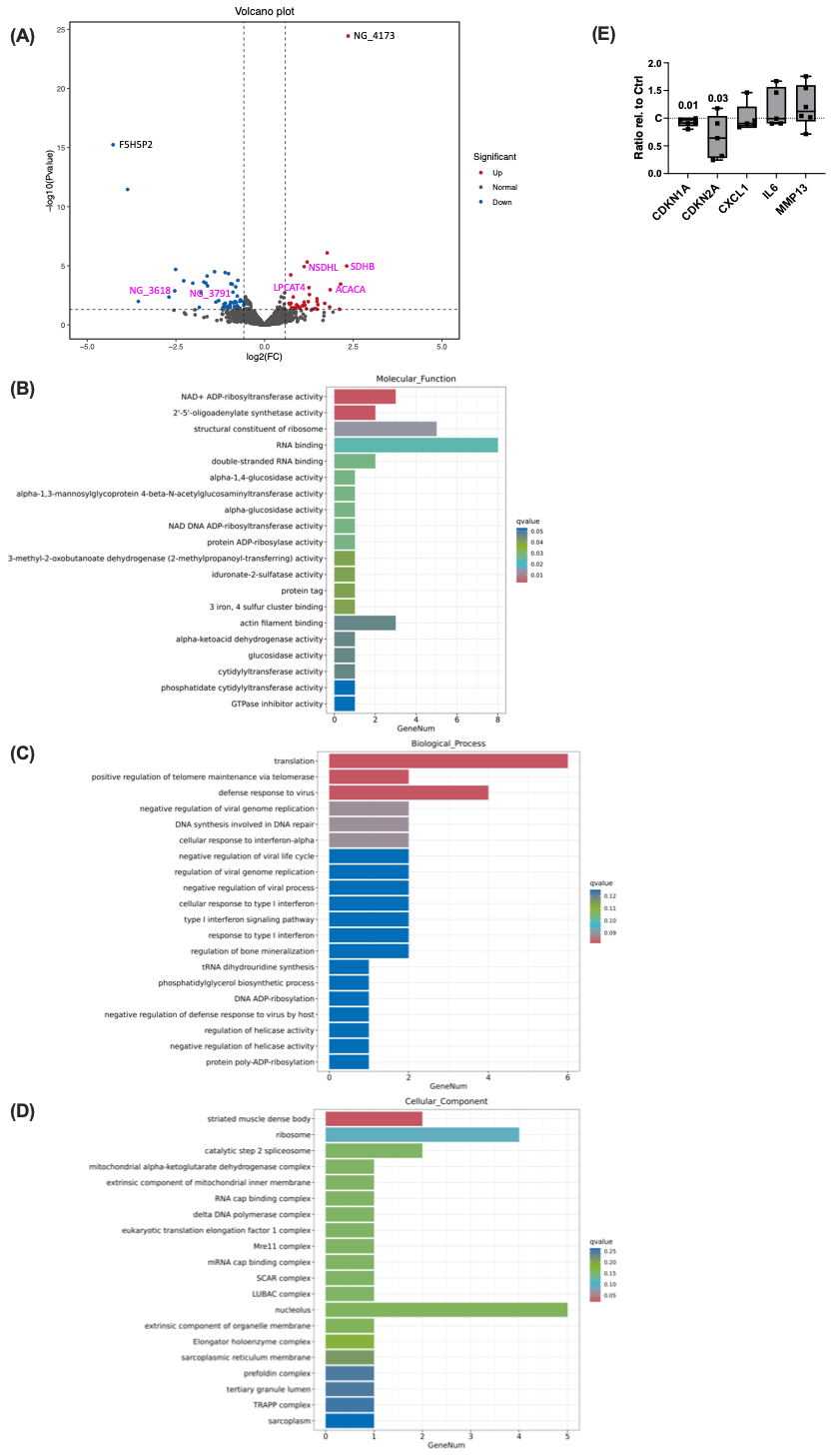
**

(A) Volcano blot of differentially expressed genes in hAC after GDF-15 stimulation. Gene names of proteins related to the glycerophospholipid metabolism are labelled in pink. (B-D) Gene Ontology (GO) enrichment of significantly regulated genes in response to rhGDF-15; top terms classified as (B) molecular function, (C) biological process, and (D) cellular components. (E) qRT-PCR analysis of senescence-associated markers 48 h after GDF-15 stimulation.

**Figure S5: Dose-dependent effects of rhGDF-15 on proliferation (alamarBlue) and migration (scratch assay) of hAC**

(A) Proliferation of hAC in response to different concentrations of rhGDF-15 was estimated by means of an alamarBlue assay. (B) Migration of hAC in response to different concentrations of rhGDF-15 was estimated by means of a scratch assay. Assessment was conducted 48h after stimulation with rhGDF-15.

**Figure S6: Influence of GDF-15 on directed (boyden chamber) and non-directed (scratch assay) migration**

(A) Boyden chamber assay of human mesenchymal stem cells in response to rhGDF-15 or PDGF as positive control (PK). Scratch assay of hAC in response to (B) rhGDF-15 w/ or w/o inhibitory TGF receptor antibody or (C) ErbB2 inhibitor.

**Figure S7: Anti-inflammatory/ immunomodulatory effects of rhGDF-15.**

Gene expression of (A) TNF, (B) IL1B, and (C) IL6 in 100 ng/ mL LPS + 20 nM IFNy-stimulated THP-1 cells w/ or w/o addition of GDF-15. Gene expression of (D) COL2A1, (E) COX2, and (F) MMP13 in IL-1ꞵ-stimulated hAC w/ or w/o addition of GDF-15.

**Table S8: List of primer and TaqMan® gene expression assays used for qRT-PCR.**

| **Target gene** | **MasterMix** | **Primer sequence/ TaqMan Probe** |
| --- | --- | --- |
| 18S rRNA | Power SYBR® Green PCR Master Mix (Applied Biosystems) | 5′-CGC​AGC​TAG​GAA​TAA​TGG​AAT​AGG-3’ (forward) and 5′-CAT​GGC​CTC​AGT​TCC​GAA​A-3’ (reverse) |
| CDKN1A (P21) | TaqMan® fast advanced MasterMix | Hs00355782 |
| CDKN2A (P16INK4/P14ARF) |  | Hs00923894 |
| COL2A1 |  | Hs00264051 |
| CXCL1 |  | Hs00605382 |
| ERBB2 |  | Hs01001580_m1 |
| GAPDH |  | Hs02758991 |
| GAPDH | Platinum® SYBR® Green qPCR SuperMix-UDG (Invitrogen, Darmstadt, Germany) | 5′-TGG​TAT​CGT​GGA​AGG​ACT​CAT​G-3’ (forward) and 5′-TCT​TCT​GGG​TGG​CAG​TGA​TG-3’ (reverse) |
| GDF15 | TaqMan® fast advanced MasterMix | Hs00171132_m1 |
| GFRAL |  | Hs01087628_m1 |
| HPRT1 |  | Hs02800695 |
| IL1B |  | Hs00174097_m1 |
| IL6 |  | Hs00985639 |
| MMP-13 |  | Hs00233992 |
| TMEM199 |  | Hs01022209 |
| TNF |  | Hs01113624_g1 |
| TP53 |  | Hs01034249_m1 |

**Figure S9: Influence of previous hyaluronidase digestion on concentrations of synovial GDF-15.**

Comparison between syGDF-15 quantification with (w/) or without (w/o) preceding hyaluronidase digestion.
